# Gabapentinoids: Gabapentin and Pregabalin for Postoperative Pain Management

**DOI:** 10.5812/aapm.7743

**Published:** 2012-09-13

**Authors:** Farnad Imani, Poupak Rahimzadeh

**Affiliations:** 1 Department of Anesthesiology and Pain Medicine, Iran University of Medical Sciences, Tehran, IR Iran

**Keywords:** Pregabalin, Gabapentin, Pain

Editorial

Postsurgical pain is normally perceived as nociceptive pain. Surgical trauma has been known to induce central and peripheral sensitization and hyperalgesia, which in untreated cases could lead to chronic postoperative pain after surgery. Indeed pain is one of the three most common medical causes of delayed discharge after ambulatory surgery, the other two being drowsiness and nausea/ vomiting. Antihyperalgesic drugs improve postoperative pain by preventing the development of central sensitization (1). The development of newer agents available for postoperative pain control create possibilities for better combinations in multimodal analgesia. The recent advances in postoperative pain management can be specifically grouped in the following areas: Finding exact molecular mechanisms, new pharmaceutical products and other routes and modes of analgesic delivery. For the years, opioids have been the mainstay of postoperative pain management but they have side effects. For this purpose the multimodal approach and non-opioid drugs have been suggested to improve postoperative analgesia and to reduce opioid related side effects (2). Gabapentinoids (gabapentin and pregabalin) were originally introduced as antiepileptics but have analgesic, anticonvulsant, and anxiolytic effects also. These easily tolerable drugs by patients have limited side-effects. Gabapentin an anti-epileptic drug binds to the alpha-2 delta subunit of the presynaptic voltage gated-calcium channels and inhibits calcium release so prevents the release of excitatory neurotransmitters involved in the pain pathways (2, 3). Gabapentin has demonstrated analgesic effect in diabetic neuropathy, post-herpetic neuralgia, and neuropathic pain. Several meta-analyses reveal that perioperative gabapentin helps to produce a significant opioid-sparing effect and probably decreses postoperative pain score relative to the control group (4, 5). Pregabalin is a structural analog of gamma-aminobutyric acid (GABA). It acts by presynaptic binding to the α -2-λ subunit of voltage-gated calcium channels that are widely distributed in the spinal cord and brain6. By this mechanism, pregabalin modulates the release of several excitatory neurotransmitters, such as glutamate, norepinephrine, substance P, and calcitonin gene-related peptide. It leads to inhibitory modulation of “overexcited” neurons and returning them to a “normal” state. Centrally, pregabalin could reduce the hyperexcitability of dorsal horn neurons that is induced by tissue damage (6). To sum up, pregabalin has a more appropriate pharmacokinetic profile than gabapentin, including dose-independent absorption and far more potent than gabapentin while producing fewer adverse effects (7-9). Pregabalin has efficacy of varying degree in neuropathic pain conditions such as postherpetic neuralgia, painful diabetic neuropathy, central neuropathic pain, and fibromyalgia. While some surveys do not demonstrate a significant analgesic effect in the acute, including postoperative pain control (9, 10), other studies suggest pregabalin to have effective sedative and opioid-sparing effects (11-13), and emphasize on its effectiveness in acute pain control. Since safe postoperative pain control is nessecary, established role of pregabalin as an analgesic adjuvant as a part of multimodal analgesia for acute pain control is in progress (7, 8, 14, 15). Its unique potency in reducing opioid requirements, prevention of opioid tolerance, enhancement the quality of opioid analgesia, decreased respiratory depression and anxiolysis, make it an attractive drug to consider for control of pain in the post-operative setting (15-17). Lots of meta-analyses and clinical trials show that perioperative pregabaline helps to produce a significant opioid-sparing effect and probably improves postoperative pain score relative to the control group (18-21). Having looked at these two drugs from different angles and aspects, one comes to this understanding that these multi-purpose drugs have found a strong and reliable place in acute pain service setting. So, Gabapentinoids are an effective tool in the treatment of postoperative pain.
